# Complicated Acute Cholecystitis: The Role of C-Reactive Protein and Neutrophil-Lymphocyte Ratio as Predictive Markers of Severity

**DOI:** 10.7759/cureus.13592

**Published:** 2021-02-27

**Authors:** Fahad Mahmood, Akinfemi Akingboye, Yogeshkumar Malam, Mehual Thakkar, Periyathambi Jambulingam

**Affiliations:** 1 General Surgery, Queen Elizabeth Hospital Birmingham, Birmingham, GBR; 2 Colorectal Surgery, Russells Hall Hospital, Dudley, GBR; 3 General Surgery, Luton & Dunstable University Hospital NHS Foundation Trust, Luton, GBR

**Keywords:** complicated acute cholecystitis, gangrenous cholecystitis, neutrophil-lymphocyte ratio, c-reactive protein

## Abstract

Objectives

The clinical diagnosis of complicated acute cholecystitis (CAC) remains difficult with several pathological or ultrasonography criteria used to differentiate it from uncomplicated acute cholecystitis (UAC). This study aims to evaluate the use of combined inflammatory markers C-reactive protein (CRP) and neutrophil-to-lymphocyte ratio (NLR) as surrogate markers to differentiate between UAC and CAC.

Methods

We identified 600 consecutive patients admitted with biliary symptoms during an acute surgical take from our electronic prospectively maintained database over a period of 55 months. Only patients undergoing emergency cholecystectomy performed during the index admission were included. The primary outcome was the finding of CAC versus UAC.

Results

A total of 176 patients underwent emergency laparoscopic cholecystectomy (ELC) during the index admission, including 118 (67%) females with a median age of 51 years (range: 21-97 years). The proportion of UAC (130 [74%]) and CAC (46 [26%]) was determined along with demographic data. Multivariate regression analysis showed that patient’s age (OR=1.047; p=0.003), higher CRP (OR=1.005; p=0.012) and NLR (OR=1.094; p=0.047) were significant independent factors associated with severity of cholecystitis. Receiver operating characteristic (ROC) analysis for CRP showed an AUC (area under the curve) of 0.773 (95% CI: 0.698- 0.849). Using a cut-off value of 55 mg/L for CRP, the sensitivity of CAC was 73.9% and specificity was 73.1% in predicting CAC. The median post-operative length of stay was four days. The conversion rate from laparoscopic cholecystectomy to open surgery was 2% (4/176), and 5% (9/176) patients suffered post-operative complications with no mortality at 30 days.

Conclusion

CRP, NLR and age were independent factors associated with the severity of acute cholecystitis. NLR and CRP can be used as surrogate markers to predict patients at risk of CAC during emergency admission, which can inform future guidelines. Moreover, ELC for CAC can be safely performed under the supervision of dedicated upper GI surgeons.

## Introduction

Each year approximately 1% to 2% of patients with asymptomatic cholelithiasis develop acute cholecystitis [1]. Gangrenous cholecystitis (GC), which affects 2-30% of all cases, is a severe form of acute cholecystitis associated with increased morbidity and a reported mortality of 15-50% [2-5]. This occurs when acute cholecystitis progresses to the advanced stage of inflammation, resulting in perforation secondary to gallbladder wall ischaemia. This ischaemia is a consequence of an impacted cystic duct causing distension of the gallbladder and increased tension on the gallbladder wall, resulting in vascular compromise accompanied by an associated inflammatory reaction [2,3,6,7]. Moreover, bacterial infection has only been identified in 50% of cases [2]. Furthermore, empyema of the gallbladder has a reported incidence of 2-11% with an associated higher risk of mortality, which can also be difficult to diagnose pre-operatively [8]. Thus, early emergency cholecystectomy is recommended in suspected cases to reduce the risk of further complications, particularly in complicated acute cholecystitis (CAC).

Early diagnosis of CAC is a challenge and is not considered until the patient has deteriorated. The 2018 Tokyo guidelines (TG18) provide parameters for the diagnosis of acute cholecystitis (physical examination, C-reactive protein [CRP], white blood cell [WBC] count and radiology) as well as risk stratification parameters including a raised WBC count of >18x10^9^/L [9,10]. Although studies have evaluated other inflammatory markers as surrogate markers for increased CAC risk, these have not gained widespread usage. We aim to evaluate whether the use of pre-operative CRP and neutrophil-lymphocyte ratio (NLR) as surrogate markers could serve to differentiate between uncomplicated acute cholecystitis (UAC) and CAC.

The preliminary data from our project was presented at the November 2015 meeting of the Association of Laparoscopic Surgeons of Great Britain and Ireland. 

## Materials and methods

A total of 600 consecutive patients admitted with acute cholecystitis over a period of 55 months (April 2010 to February 2015) at a single UK district general hospital were identified; 176 patients undergoing emergency laparoscopic cholecystectomy (ELC) during the index admission were analysed as part of a prospective cohort study. Diagnosis of acute cholecystitis was confirmed with clinical features (right upper quadrant pain, fever and Murphy’s sign), inflammatory markers WBC as well as CRP, and departmental ultrasound scan (USS) showing gallstones, thickened gallbladder and/or peri-cholecystic fluid. Routine full blood count, renal and liver function tests and clotting profile were obtained on admission for all patients. Histological evaluation post-operatively was used to confirm an intra-operative diagnosis of gangrene.

All patients above aged 18 years without contraindication to ELC were eligible for inclusion. Those with common bile duct (CBD) stones, cholangitis and pancreatitis, as well as pregnant patients were excluded. Similarly, patients on steroid use, those with human immunodeficiency virus (HIV), leukaemia or other haematological malignancy, and those on cytotoxic chemotherapy were excluded due to the possibility of affecting WBC differential. All patients undergoing ELC received intravenous antibiotics and treatment by the same experienced surgical team comprising four consultant upper GI surgeons. Laparoscopic cholecystectomy was performed using the standard four-port technique, with the operating surgeon standing on the left of the patient. Patients were classified intra-operatively into those with UAC and CAC. CAC was defined as the presence of gallbladder empyema, necrosis (patchy or complete), gangrene or perforation to differentiate it from UAC.

Data collected included demographics and clinical parameters such as age, gender, length of hospital stay, time until the surgery, CRP, WBC, NLR, liver function and renal function, as well as seniority of primary operating surgeon (consultant vs trainee). Procedures performed by trainee registrars were under the direct supervision of the consultant surgeon. The primary endpoint was the comparison of CRP and NLR in predicting intra-operative CAC. Receiver operating characteristic (ROC) curve analysis as well as multivariable logistic regression analysis were used to determine factors predicting the severity of cholecystitis. All statistical analysis was performed using IBM SPSS Statistics software program, Version 25.0 (IBM Corp., Armonk, NY, USA). A p-value of ≤0.05 was considered statistically significant.

No ethical approval was required for the conduct of this observational study from our institution as it was registered as a quality improvement project with our local audit department. The reporting of the study has been in accordance with the STROBE (Strengthening the Reporting of Observational Studies in Epidemiology) guidelines.

## Results

Study population

A total of 176 patients underwent ELC. The median age was 51 years (range: 21-97 years), of which 118 (67%) were female and 58 (33%) male. The mean WBC count was 12.6x10^9^/L (SD: 5.1-30x10^9^/L) and CRP was 88.4 mg/L (SD: 117.5 mg/L) on admission. Furthermore, the mean NLR was 9.38 (SD: 8.76). For liver function, mean bilirubin was 20.94 μmol/L (SD: 23.4 μmol/L), mean alanine transaminase (ALT) was 62.31 IU/L (SD: 91.42 IU/L) and mean alkaline phosphatase (ALP) was 96.55 IU/L (SD: 79.70IU/L). Mean creatinine was 79.04 μmol/L (SD: 30.88 μmol/L) (Table 1). Radiological confirmation of the diagnosis was obtained in all cases. A thick-walled gallbladder (≥3 mm) was confirmed on USS in 134 (76%) patients, with 34 (19%) patients showing evidence of peri-cholecystic fluid (Table 2). Median delay in surgery from time of admission was two days (range: 0-16 days) and median length of stay post-operatively was four days (range: 0-32 days) (Table 1). Sixteen (9%) patients underwent surgery on the same day, with 51 (28.9%) undergoing surgery within one day. Only three patients waited longer than seven days for an operation, of which two had CAC.

**Table 1:** Summary of patient demographics, delay in treatment, length of stay, average inflammatory markers, and liver and renal function. A total of 176 patients were included, with 118 females and 58 males. WBC, white blood cell; NLR, neutrophil-to-lymphocyte ratio; CRP, C-reactive protein

	Average	Range	±Standard Deviation
Age	51 years	21-97 years	17.63
Time until surgery	2 days	0-16 days	2.04
Length of stay	4 days	0-32 days	4.03
WBC	12.6x10^9^/L	5.1-30x10^9^/L	4.66x10^9^/L
Neutrophil count	10.15x10^9^/L	2.70-28.86x10^9^/L	4.53x10^9^/L
Lymphocyte count	1.60x10^9^/L	0.20-5.27x10^9^/L	0.89x10^9^/L
NLR	9.38	1.16-52.47	8.76
CRP	88.4 mg/L	1-500 mg/L	117.5 mg/L
Bilirubin	20.94 μmol/L	3-237	23.40
Alanine transaminase	62.31 IU/L	11-469	91.42
Alkaline phosphatase	96.55 IU/L	25-762	79.70
Creatinine	79.04 μmol/L	4-248	30.88

**Table 2:** Ultrasonographic findings in patients presenting with acute cholecystitis. The mean gallbladder wall thickness was 4.5 (SD±2.6) mm. A cut-off value of 3 mm was used to define a thick-walled gallbladder.

	Average	± Standard Deviation
Thick-wall gallbladder (≥ 3 mm)	134 (≥ 3 mm)	42 (≤2 mm)
Peri-cholecystic fluid	Present in 34	Absent in 142

**Figure 1:**
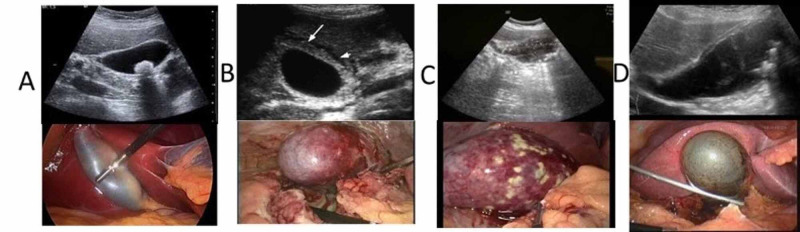
Ultrasonographic findings and the corresponding intra-operative images of (A) normal gallbladder, (B) acute cholecystitis, (C) gallbladder empyema and (D) gangrenous cholecystitis.

Four upper GI consultants performed all operations or supervised registrars (residents) in performing these (44/176). There were 4/176 (2%) cases of laparoscopic cholecystectomy converted to open surgery. Intra-operative confirmation of GC in 30 (17%) and gallbladder empyema in 16 (9%) patients, which constitute CAC, was obtained (Table 3). The correlation between radiological and intra-operative findings is illustrated in Figure 1. Furthermore, 9/176 (5%) patients suffered post-operative complications including four cases of bile leak: three were managed with endoscopic retrograde cholangiopancreatography (ERCP), whereas one required re-laparoscopy, washout and over-sewing of an accessory duct of Luschka. Moreover, three cases of subhepatic collection were managed by ultrasound-guided drainage. Four patients developed systemic sepsis requiring intravenous antibiotics and high dependency unit admission, two with UAC and two with CAC. One patient required T-tube insertion into the CBD following exploration and duct clearance. There was no reported mortality at 30 days and 90 days’ follow-up. Histological confirmation of GC was confirmed in 36 (20.4%) patients (Table 3).

**Table 3:** Intra-operative and histological findings of patients presenting with acute cholecystitis.

	Intra-operative	Histology
Acute cholecystitis	130	140
Gallbladder empyema	16	-
Gangrenous cholecystitis	30	36

Predictive factors for complicated acute cholecystitis

Intra-operative findings showed UAC in 130 (74%) patients, with 46 (26%) showing evidence of CAC (Table 3). Multivariate regression analysis showed that increasing age (p=0.003; OR=1.047; 95% CI: 1.016-1.080), CRP (p=0.012’ OR=1.005; 95% CI: 1.001-1.009) and NLR (p=0.047; OR=1.094; 95% CI: 1.001-1.196) were significantly associated with a higher risk of CAC. Furthermore, gender, time until surgery and grade of primary operating surgeon was not associated with higher risk of CAC (Table 4). Other biochemical markers including WBC count, neutrophils, lymphocytes, bilirubin, ALT, ALP and creatinine did not show a significant association with the severity of cholecystitis (Table 4). Moreover, radiological parameters including gallbladder wall thickness and the presence of peri-cholecystic fluid were not associated with CAC. The variability predicted by this regression model was 41.5% (Nagelkerke r2). The Hosmer-Lemeshow test predicting poor model fitness was not significant (p=0.733). In addition, no clinical, biochemical, or radiological parameters were predictive of a histological evidence of gangrene (Table 5).

**Table 4:** Logistic regression analysis for factors associated with an intra-operative diagnosis of complicated acute cholecystitis. Increasing age and rising CRP showed significant association with intra-operative diagnosis of complicated acute cholecystitis. *Statistical significance of p<0.05. CRP, C-reactive protein; WBC, white blood cell; NLR, neutrophil-to-lymphocyte ratio

	Odds ratio (95% CI)	p-Value
Age	1.047 (1.016-1.080)	0.003*
Gender	0.729 (0.283-1.882)	0.514
CRP	1.005 (1.001-1.009)	0.012*
WBC count	1.473 (0.752-2.885)	0.259
Neutrophils	0.678 (0.331-1.390)	0.289
Lymphocytes	0.985 (0.346-2.804)	0.977
NLR	1.094 (1.001-1.196)	0.047*
Bilirubin	1.012 (0.990-1.034)	0.282
Alanine transaminase	1.002 (0.994-1.009)	0.676
Alkaline phosphatase	0.992 (0.980-1.004)	0.200
Creatinine	0.989 (0.966-1.013)	0.371
Delay in surgery	0.881 (0.700-1.111)	0.284
Radiological thickness of the gallbladder wall	0.463 (0.122-1.752)	0.257
Radiological peri-cholecystic fluid	6.960 (0.453-106.885)	0.164
Surgeon grade	0.539 (0.184-1.580)	0.260

**Table 5:** Logistic regression analysis of factors associated with histological diagnosis of gangrenous cholecystitis showed only increasing age as a risk factor. CRP, C-reactive protein; WBC, white blood cell; NLR, neutrophil-to-lymphocyte ratio

	Odds ratio (95% CI)	p-Value
Age	1.055 (1.010-1.101)	0.829
Gender	0.745 (0.219-2.534)	0.638
CRP	1.003 (0.999-1.008)	0.112
WBC count	1.156 (0.310-4.310)	0.829
Neutrophils	0.916 (0.224-3.746)	0.903
Lymphocytes	0.641 (0.103-3.992)	0.634
NLR	0.978 (0.881-1.086)	0.678
Bilirubin	0.994 (0.959-1.030)	0.750
Alanine transaminase	0.995 (0.983-1.006)	0.371
Alkaline phosphatase	0.999 (0.987-1.012)	0.896
Creatinine	1.008 (0.983-1.033)	0.553
Delay in surgery	0.918 (0.728-1.157)	0.470
Radiological thickness of the gallbladder wall	0.000 (0.000-0.000)	0.998
Radiological peri-cholecystic fluid	0.515 (0.132-2.013)	0.338
Surgeon grade	0.514 (0.132-2.004)	0.260

ROC of inflammatory markers for predicting complicated acute cholecystitis

**Figure 2:**
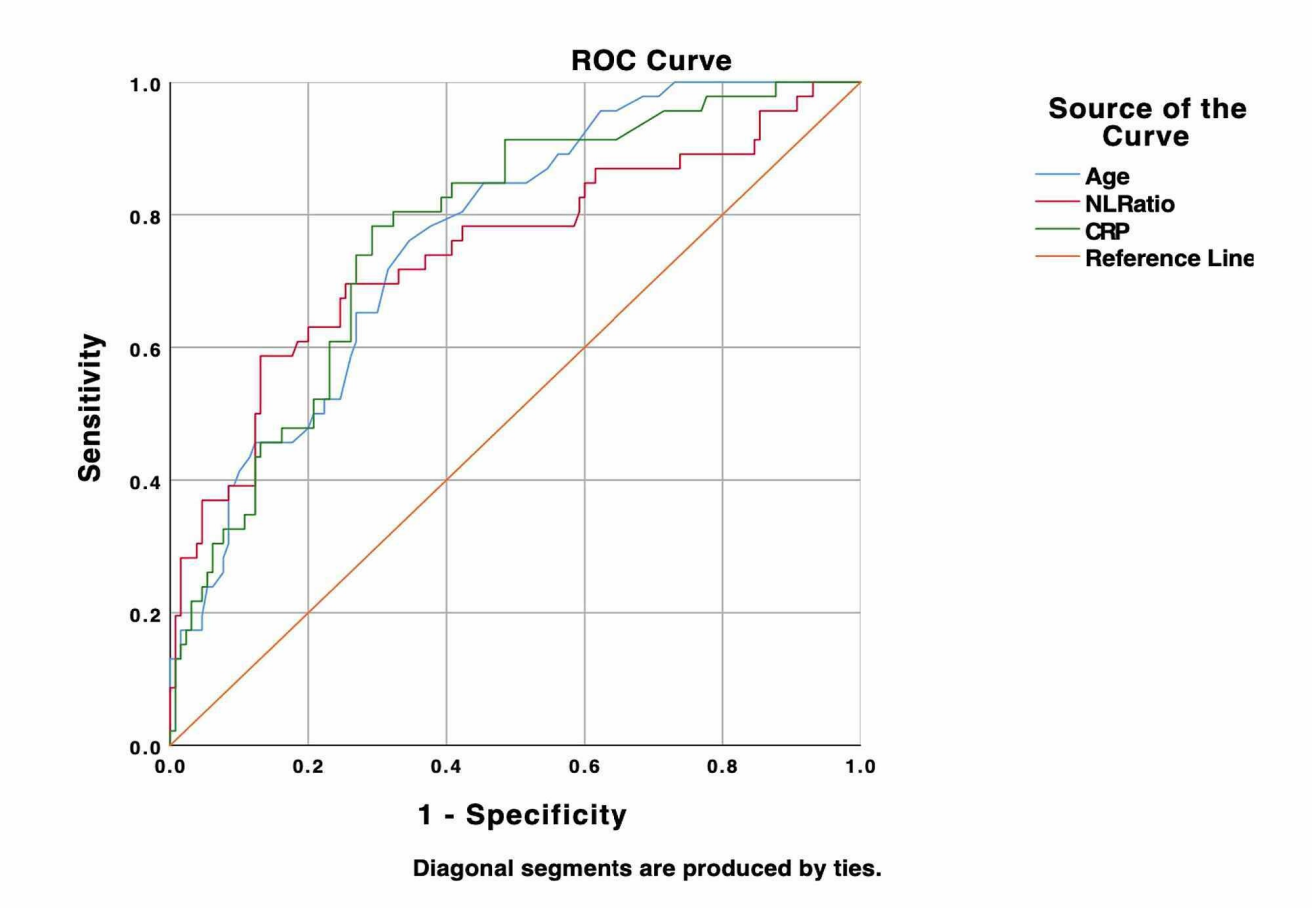
ROC curve for factors predicting complicated acute cholecystitis. For CRP, the AUC was 0.773 (95% CI 0.698-0.849). For NLR, the AUC was 0.746 (95% CI 0.655-0.837). AUC, area under the curve; CRP, C-reactive protein; NLR, neutrophil-to-lymphocyte ratio; ROC, receiver operating characteristic

ROC curves for inflammatory markers including CRP and NLR were generated to determine their predictive value for CAC (Figure 2). For CRP, the area under the curve (AUC) was 0.773 (95% CI: 0.698- 0.849). Using a cut-off value of 55 mg/L for CRP, the sensitivity of CAC was 73.9% and specificity was 73.1%. In addition, for NLR, the AUC was 0.746 (95% CI: 0.655-0.837). Using a cut-off value of 8 for NLR, the sensitivity for CAC was 71.7% and specificity of 66.9%.

## Discussion

Our study investigated early severity markers predicting CAC with a view to help determine early intervention and reduce risk of complications. Our results advocate a CRP greater than 55 mg/L and a raised NLR as significant independent predictive factors for CAC, along with increasing age. There were no overall differences in morbidity, mortality or length of stay between patients with UAC and CAC. Moreover, delay in surgery did not predict greater severity or worse outcomes. In contrast to previous studies, bilirubin and other liver function tests were not predictors of severity [11,12]. Commonly used radiological parameters such as gallbladder wall thickness and the presence of peri-cholecystic fluid were also poor predictors of intra-operative severity. Therefore, CRP and NLR should be considered additional parameters for assessing pre-operative risk of CAC.

GC was first reported by Hotchkiss in 1894 and later elucidated as a progressive pathological formation by Morfin et al. in 1968 [13,14]. The principal cause is the result of an impacting gallstone obstructing the cystic duct. In about 80% of cases, the stone dislodges itself, permitting conservative non-operative management of patients [15]. However, due to severity of the condition, laparoscopic surgery is challenging, with reported conversion rates to open surgery ranging from 2% to 10% [12,16]; recent studies by Hunt et al. [2] and Ӧnder et al. [4] reported conversion rates of 8.7% and 14%, respectively. Furthermore, the risk of conversion decreases with increasing consultant caseload [16]. Our study suggests that a dedicated consultant-led and supervised operation can significantly reduce the conversion rate (2%), which is lower than the published series. However, this may be explained by the greater proportion of females in our cohort, with male gender associated with difficult laparoscopic cholecystectomies [17]. Nonetheless, early identification of high-risk patients can expedite intervention as well as plan appropriate surgical resources.

Several studies have attempted to determine predictive factors for GC by assessing pre-operative risk factors. These include clinical, biochemical and radiological parameters, with some going further to develop scoring systems. Clinically, male gender, advanced age (greater than 50 years), African-American race, cardiovascular disease and diabetes have been shown to correlate with increased risk of GC and CAC [3,11]. Although in the present study we found advancing age to be a risk factor for CAC, we do not report a gender association possibly due to a greater proportion of females (67%) in our dataset. In addition, combinations of inflammatory markers have been investigated for association with GC. Leucocytosis with WBC count varyingly greater than 17x10^9^/L [18], 13x10^9^/L [19] or 14.9x10^9^/L [3] correlate with GC [3,5,6]. NLR has also been used as a potential marker of severe cholecystitis [20,21]. Micić et al. showed NLR above 4.18 with 78.3% sensitivity and 74.3% specificity to predict severe cholecystitis [22]. Moreover, Lee et al. showed an NLR of >3.0 to distinguish simple cholecystitis from severe cholecystitis, as well as functioning as predicting increased length of stay [23]. Lee et al. further showed NLR above 3.0 to have a 70.5% sensitivity and 70% specificity in predicting severe cholecystitis [23]. In addition, CRP has also shown promise as a predictor of severity in acute cholecystitis. Nikfarjam et al. identified GC with a CRP of ≥94 mg/L compared with 17 mg/L for non-GC [7]. Juvonen et al. demonstrated elevated CRP associated with both infected bile and GC [24]. Mok et al. further found that a CRP value greater than 200 mg/dL has a 50% positive predictive value and 100% negative predictive value, with 100% sensitivity and 87.9% specificity, in predicting GC [25]. Real-Noval et al. demonstrated an AUC of 0.872 with a CRP cut-off of 152.5 mg/L showing 91% sensitivity and 70% specificity for GC [21]. More recent studies have further corroborated the role of CRP as a discriminator for CAC, with AUC of 0.75 and a cut-off of 60.5 mg/L showing 71% sensitivity and 71.4% specificity [26]. Interestingly, CRP in this study also predicted increased risk of open conversion. In addition, other biochemical parameters have been investigated for predicting GC. Fagan et al. found that elevated AST (aspartate transaminase) and ALT were not predictive of GC but rather indicative of hepatic necrosis [3]. Others have varyingly shown elevated bilirubin, urea and creatinine as factors associate with CAC, but these results have not been widely replicated [7,12]. In summary, our study investigated routinely tested haematological and biochemical markers for predicting severity that can be assessed by all surgeons. Although leucocytosis did not reach statistical significance, we were able to show the discriminative value of NLR ratio with a cut-off value of 8. Moreover, consistent with the above studies, we have shown CRP to have significant value in predicting CAC with a cut-off value of 55 mg/L and advocate its use beyond a diagnostic marker for acute cholecystitis as advocated by. Our findings may help determine patients with CAC who may benefit from immediate ELC. We have not fully explored all the possible variables that may predict the development of severity, morbidity or mortality, although we did not find liver function tests to be predictive.

There are multiple radiological studies characterising sonographic features attributed to GC. These include gallbladder wall thickening, gallbladder wall striations, gallbladder wall irregularities, intraluminal membranes, complex peri-cholecystic fluid collections and the absence of Murphy sign [27]. However, appreciating that these features can overlap with those found in acute cholecystitis, a study by Teefey et al. argued that peri-cholecystic fluid, gallbladder wall striation and negative Murphy sign were not predictive factors for GC but gallbladder wall thickness was [27]. In addition, Bennett et al. reviewed the diagnostic features of GC illustrated by computed tomography (CT) scan [28]. They concluded that air in the gallbladder wall or lumen, irregular or absent gallbladder wall, intraluminal membranes, peri-cholecystic abscess and an absence of gallbladder wall enhancement, along with pronounced gallbladder wall thickening and distention of the short axis are specific CT features suggestive of GC [28]. In our practice, CT scan is dedicated to investigating an acute abdomen or in cases of diagnostic uncertainty in patients with right-sided abdominal pain. In the present study, we did not demonstrate the correlation of specific USS findings including gallbladder wall thickness or the presence of peri-cholecystic fluid with increased risk of CAC. The relative numbers in our study and the single operator dependency of USS may limit interpretation of what may be promising prognostic markers to explore in future studies.

Finally, studies have attempted to develop scoring systems for assessing the severity of acute cholecystitis. Yacoub et al. developed a scoring system to ascertain the probability of GC based on variables including age >45 years (1 point), male (2 points), WBC count > 13x10^9^/L (1.5 points), ultrasound gallbladder wall thickening of 4.5 mm (1 point) and heart rate of >90 beats/minute (1 point) [29]. In this study, GC had an 87% prevalence in the high probability category (4.5 points) of patients. A further study by Wu et al. validated the Yacoub scoring system, with only gender losing significance in the multivariate analysis [19]. They adjusted the model creating a five-point scoring system with five points yielding a 63% probability of GC [29]. Based on increased data supporting the use of CRP and NLR as severity markers, further studies are needed to incorporate and validate these in scoring systems in prospective multicentre trials.

Limitations of our study include the relatively small sample size, role of early intravenous antibiotics in treating acute cholecystitis and lack of consideration of significant comorbidities. Furthermore, we did not explore the use of other acute phase reactants such as procalcitonin in predicting severe acute cholecystitis.

## Conclusions

CRP, NLR and age were found to be independent factors associated with severity of acute cholecystitis and able to predict CAC. Inflammatory markers are a valuable adjunct to the assessment of severity in acute cholecystitis and planning appropriate management. Despite the challenges involved in performing ELC for severe CAC, our study has shown that it can be safely performed under the supervision of dedicated upper GI surgeons. Validation of these findings in the emergency setting is required with larger prospective multicentre trials to support decision-making and resource allocation for ELC.
